# Supplementary material for: Serial Measurements of Circulating KL-6, SP-D, MMP-7, CA19-9, CA-125, CCL18, and Periostin in Patients with Idiopathic Pulmonary Fibrosis Receiving Antifibrotic Therapy: An Exploratory Study
Source: J Clin Med. 2021 Aug 28;10(17):3864. doi: 10.3390/jcm10173864 (PMC8432145; doi:10.3390/jcm10173864)
Supplement: Supplementary file 1 [file jcm-10-03864-s001.zip › jcm-1293525-supplementary.pdf]

Supplementary Material

Table S1. Correlations between serum biomarkers levels in IPF patients at baseline.

	KL-6 (U/mL)	SP-D (ng/mL)	CA19-9 (U/mL)	CA-125 (U/mL)	MMP-7 (ng/mL)	Periostin (ng/mL)	CCL18 (ng/mL)
**KL-6 (U/mL)**		r = 0.49<br>*p* = 0.008	r = 0.20<br>*p* = 0.32	r = -0.18<br>*p* = 0.37	r = -0.01<br>*p* = 0.98	r = -0.03<br>*p* = 0.87	r = 0.44<br>*p* = 0.02
**SP-D (ng/mL)**	r = 0.49<br>*p* = 0.008		r = -0.03<br>*p* = 0.87	r = -0.52<br>*p* = 0.005	r = -0.01<br>*p* = 0.95	r = -0.15<br>*p* = 0.44	r = -0.02<br>*p* = 0.92
**CA19-9 (U/mL)**	r = 0.20<br>*p* = 0.32	r = -0.03<br>*p* = 0.87		r = 0.18<br>*p* = 0.35	r = 0.10<br>*p* = 0.60	r = -0.11<br>*p* = 0.57	r = 0.09<br>*p* = 0.65
**CA-125 (U/mL)**	r = -0.18<br>*p* = 0.37	r = -0.52<br>*p* = 0.005	r = 0.18<br>*p* = 0.35		r = 0.36<br>*p* = 0.06	r = 0.30<br>*p* = 0.12	r = 0.15<br>*p* = 0.44
**MMP-7 (ng/mL)**	r = -0.01<br>*p* = 0.98	r = -0.01<br>*p* = 0.95	r = 0.10<br>*p* = 0.60	r = 0.36<br>*p* = 0.06		r = 0.29<br>*p* = 0.14	r = -0.16<br>*p* = 0.41
**Periostin (ng/mL)**	r = -0.03<br>*p* = 0.87	r = -0.15<br>*p* = 0.44	r = -0.11<br>*p* = 0.57	r = 0.30<br>*p* = 0.12	r = 0.29<br>*p* = 0.14		r = 0.25<br>*p* = 0.20
**CCL18 (ng/mL)**	r = 0.44<br>*p* = 0.02	r = -0.02<br>*p* = 0.92	r = 0.09<br>*p* = 0.65	r = 0.15<br>*p* = 0.44	r = -0.16<br>*p* = 0.41	r = 0.25<br>*p* = 0.20	

Abbreviations: KL-6 – Krebs von den Lungen-6, SP-D – surfactant protein D, CA19-9 – cancer antigen 19-9, CA-125 – cancer antigen 125, MMP-7 – matrix metalloproteinase 7, CCL18 – chemokine (C-C motif) ligand 18.

Table S2. Correlations between serum biomarkers levels in IPF patients after 6 months of antifibrotic therapy.

	KL-6 (U/mL)	SP-D (ng/mL)	CA19-9 (U/mL)	CA-125 (U/mL)	MMP-7 (ng/mL)	Periostin (ng/mL)	CCL18 (ng/mL)
**KL-6 (U/mL)**		r = 0.46<br>*p* = 0.01	r = 0.17<br>*p* = 0.40	r = -0.05<br>*p* = 0.82	r = -0.15<br>*p* = 0.45	r = 0.08<br>*p* = 0.67	r = 0.08<br>*p* = 0.68
**SP-D (ng/mL)**	r = 0.46<br>*p* = 0.01		r = 0.05<br>*p* = 0.82	r = -0.53<br>*p* = 0.004	r = -0.08<br>*p* = 0.70	r = -0.11<br>*p* = 0.57	r = -0.24<br>*p* = 0.22
**CA19-9 (U/mL)**	r = 0.17<br>*p* = 0.40	r = 0.05<br>*p* = 0.82		r = 0.20<br>*p* = 0.31	r = 0.16<br>*p* = 0.42	r = 0.20<br>*p* = 0.31	r = -0.13<br>*p* = 0.52
**CA-125 (U/mL)**	r = -0.05<br>*p* = 0.82	r = -0.53<br>*p* = 0.004	r = 0.20<br>*p* = 0.31		r = 0.12<br>*p* = 0.55	r = -0.01<br>*p* = 0.95	r = 0.25<br>*p* = 0.19
**MMP-7 (ng/mL)**	r = -0.15<br>*p* = 0.50	r = -0.08<br>*p* = 0.70	r = 0.16<br>*p* = 0.42	r = 0.12<br>*p* = 0.55		r = 0.16<br>*p* = 0.41	r = -0.33<br>*p* = 0.09
**Periostin (ng/mL)**	r = 0.08<br>*p* = 0.67	r = -0.11<br>*p* = 0.57	r = 0.20<br>*p* = 0.31	r = -0.01<br>*p* = 0.95	r = 0.16<br>*p* = 0.41		r = 0.14<br>*p* = 0.47
**CCL18 (ng/mL)**	r = 0.08<br>*p* = 0.68	r = -0.24<br>*p* = 0.22	r = -0.13<br>*p* = 0.52	r = 0.25<br>*p* = 0.19	r = -0.33<br>*p* = 0.09	r = 0.14<br>*p* = 0.47	

Abbreviations: KL-6 – Krebs von den Lungen-6, SP-D – surfactant protein D, CA19-9 – cancer antigen 19-9, CA-125 – cancer antigen 125, MMP-7 – matrix metalloproteinase 7, CCL18 – chemokine (C-C motif) ligand 18.

Table S3. Correlations between serum biomarkers levels in IPF patients after 12 months of antifibrotic therapy.

	KL-6 (U/mL)	SP-D (ng/mL)	CA19-9 (U/mL)	CA-125 (U/mL)	MMP-7 (ng/mL)	Periostin (ng/mL)	CCL18 (ng/mL)
**KL-6 (U/mL)**		r = 0.33<br>*p* = 0.08	r = 0.08<br>*p* = 0.69	r = 0.14<br>*p* = 0.47	r = 0.12<br>*p* = 0.54	r = 0.22<br>*p* = 0.26	r = 0.23<br>*p* = 0.25
**SP-D (ng/mL)**	r = 0.33<br>*p* = 0.08		r = 0.01<br>*p* = 0.96	r = -0.45<br>*p* = 0.02	r = -0.22<br>*p* = 0.26	r = -0.06<br>*p* = 0.76	r = -0.31<br>*p* = 0.11
	r = 0.08	r = 0.01		r = 0.30	r = -0.05	r = -0.04	r = 0.20

**CA19-9 (U/mL)**	*p* = 0.69	*p* = 0.96		*p* = 0.12	*p* = 0.81	*p* = 0.86	*p* = 0.31
**CA-125 (U/mL)**	r = 0.14<br>*p* = 0.47	r = -0.45<br>*p* = 0.02	r = 0.30<br>*p* = 0.12		r = 0.33<br>*p* = 0.09	r = 0.15<br>*p* = 0.46	r = 0.45<br>*p* = 0.02
**MMP-7 (ng/mL)**	r = 0.12<br>*p* = 0.54	r = -0.22<br>*p* = 0.26	r = -0.05<br>*p* = 0.81	r = 0.33<br>*p* = 0.09		r = 0.31<br>*p* = 0.11	r = -0.05<br>*p* = 0.81
**Periostin (ng/mL)**	r = 0.22<br>*p* = 0.26	r = -0.06<br>*p* = 0.76	r = -0.04<br>*p* = 0.86	r = 0.15<br>*p* = 0.46	r = 0.31<br>*p* = 0.11		r = 0.02<br>*p* = 0.92
**CCL18 (ng/mL)**	r = 0.23<br>*p* = 0.25	r = -0.31<br>*p* = 0.11	r = 0.20<br>*p* = 0.31	r = 0.45<br>*p* = 0.02	r = -0.05<br>*p* = 0.81	r = 0.02<br>*p* = 0.92	

Abbreviations: KL-6 – Krebs von den Lungen-6, SP-D – surfactant protein D, CA19-9 – cancer antigen 19-9, CA-125 – cancer antigen 125, MMP-7 – matrix metalloproteinase 7, CCL18 – chemokine (C-C motif) ligand 18.

Table S4. Correlations between serum biomarkers levels in IPF patients after 18 months of antifibrotic therapy.

	**KL-6 (U/mL)**	**SP-D (ng/mL)**	**CA19-9 (U/mL)**	**CA-125 (U/mL)**	**MMP-7 (ng/mL)**	**Periostin (ng/mL)**	**CCL18 (ng/mL)**
**KL-6 (U/mL)**		r = 0.23<br>*p* = 0.23	r = 0.12<br>*p* = 0.53	r = 0.15<br>*p* = 0.46	r = 0.23<br>*p* = 0.24	r = 0.30<br>*p* = 0.13	r = 0.27<br>*p* = 0.16
**SP-D (ng/mL)**	r = 0.23<br>*p* = 0.23		r = -0.04<br>*p* = 0.84	r = -0.48<br>*p* = 0.01	r = 0.11<br>*p* = 0.58	r = -0.10<br>*p* = 0.61	r = -0.23<br>*p* = 0.25
**CA19-9 (U/mL)**	r = 0.12<br>*p* = 0.53	r = -0.04<br>*p* = 0.84		r = 0.23<br>*p* = 0.23	r = -0.02<br>*p* = 0.92	r = -0.03<br>*p* = 0.89	r = 0.002<br>*p* = 0.99
**CA-125 (U/mL)**	r = 0.15<br>*p* = 0.46	r = -0.48<br>*p* = 0.01	r = 0.23<br>*p* = 0.23		r = 0.21<br>*p* = 0.29	r = 0.04<br>*p* = 0.84	r = 0.10<br>*p* = 0.62
**MMP-7 (ng/mL)**	r = 0.23<br>*p* = 0.24	r = 0.11<br>*p* = 0.58	r = -0.02<br>*p* = 0.92	r = 0.21<br>*p* = 0.29		r = 0.22<br>*p* = 0.26	r = -0.41<br>*p* = 0.03
**Periostin (ng/mL)**	r = 0.30<br>*p* = 0.13	r = -0.10<br>*p* = 0.61	r = -0.03<br>*p* = 0.89	r = 0.04<br>*p* = 0.84	r = 0.22<br>*p* = 0.26		r = 0.19<br>*p* = 0.35
**CCL18 (ng/mL)**	r = 0.27<br>*p* = 0.16	r = -0.23<br>*p* = 0.25	r = 0.002<br>*p* = 0.99	r = 0.10<br>*p* = 0.62	r = -0.41<br>*p* = 0.03	r = 0.19<br>*p* = 0.35	

Abbreviations: KL-6 – Krebs von den Lungen-6, SP-D – surfactant protein D, CA19-9 – cancer antigen 19-9, CA-125 – cancer antigen 125, MMP-7 – matrix metalloproteinase 7, CCL18 – chemokine (C-C motif) ligand 18.

Table S5. Correlations between serum biomarkers levels in IPF patients after 24 months of antifibrotic therapy.

	**KL-6 (U/mL)**	**SP-D (ng/mL)**	**CA19-9 (U/mL)**	**CA-125 (U/mL)**	**MMP-7 (ng/mL)**	**Periostin (ng/mL)**	**CCL18 (ng/mL)**
**KL-6 (U/mL)**		r = 0.37<br>*p* = 0.06	r = 0.11<br>*p* = 0.57	r = 0.01<br>*p* = 0.95	r = 0.08<br>*p* = 0.70	r = 0.19<br>*p* = 0.33	r = 0.29<br>*p* = 0.13
**SP-D (ng/mL)**	r = 0.37<br>*p* = 0.06		r = 0.24<br>*p* = 0.23	r = -0.49<br>*p* = 0.008	r = -0.07<br>*p* = 0.74	r = 0.10<br>*p* = 0.61	r = -0.09<br>*p* = 0.65
**CA19-9 (U/mL)**	r = 0.11<br>*p* = 0.57	r = 0.24<br>*p* = 0.23		r = 0.11<br>*p* = 0.56	r = 0.05<br>*p* = 0.79	r = 0.06<br>*p* = 0.78	r = 0.13<br>*p* = 0.52
**CA-125 (U/mL)**	r = 0.01<br>*p* = 0.95	r = -0.49<br>*p* = 0.008	r = 0.11<br>*p* = 0.56		r = -0.06<br>*p* = 0.75	r = -0.01<br>*p* = 0.97	r = 0.35<br>*p* = 0.06
**MMP-7 (ng/mL)**	r = 0.08<br>*p* = 0.70	r = -0.07<br>*p* = 0.74	r = 0.05<br>*p* = 0.79	r = -0.06<br>*p* = 0.75		r = 0.13<br>*p* = 0.52	r = -0.23<br>*p* = 0.24
**Periostin (ng/mL)**	r = 0.19<br>*p* = 0.33	r = 0.10<br>*p* = 0.61	r = 0.06<br>*p* = 0.78	r = -0.01<br>*p* = 0.97	r = 0.13<br>*p* = 0.52		r = 0.09<br>*p* = 0.67
**CCL18 (ng/mL)**	r = 0.29<br>*p* = 0.13	r = -0.09<br>*p* = 0.65	r = 0.13<br>*p* = 0.52	r = 0.35<br>*p* = 0.06	r = -0.23<br>*p* = 0.24	r = 0.09<br>*p* = 0.67	

Abbreviations: KL-6 – Krebs von den Lungen-6, SP-D – surfactant protein D, CA19-9 – cancer antigen 19-9, CA-125 – cancer antigen 125, MMP-7 – matrix metalloproteinase 7, CCL18 – chemokine (C-C motif) ligand 18

Table S6. Longitudinal changes in PFTs, 6MWT, and circulating biomarkers concentrations in patients with IPF.

	**Baseline**	**6 months**	**12 months**	**18 months**	**24 months**
FEV_1 (l), mean (SD)	2.13 (0.60)	2.13 (0.54)	2.09 (0.56)	2.08 (0.55)	2.06 (0.56)
FEV_1% pred., mean (SD)	78.95 (19.04)	78.96 (17.82)	78.71 (18.27)	78.24 (18.57)	78.12 (18.97)
FVC (l), mean (SD)	2.65 (0.85)	2.67 (0.79)	2.59 (0.82)	2.58 (0.81)	2.56 (0.81)
FVC% pred., mean (SD)	74.95 (19.28)	75.25 (18.15)	74.16 (18.87)	74.03 (19.98)	73.78 (19.15)
FEV_1/FVC%, mean (SD)	81.53 (6.94)	80.92 (7.37)	82.10 (7.39)	82.31 (7.30)	83.84 (10.81)
$T_{L,CO}$ (mmol/min/kPa), mean (SD)	3.98 (1.08)	3.83 (1.17)	3.37 (1.24) ****§§§	3.23 (1.24) ^^^^••••	3.31 (1.14) ####±±
$T_{L,CO}$ (% of predicted), mean (SD)	53.28 (12.78)	52.56 (15.28)	45.07 (15.82) ***§§	46.92 (20.08) ^	49.47 (19.67)
6MWT (meters), mean (SD)	396.30 (104.80)	416.20 (105.3)	374.70 (126.3)	381.90 (103.6)	346.00 (110.7) # ±±
KL-6 (U/mL), median (IQR)	1277 (727.8–1755)	1643 (714.80–2078)	1494 (875.30–2735)	1723 (970.30–2595)	1490 (798.80–2565)
SP-D (ng/mL), median (IQR)	366.70 (188.8–512.7)	353.30 (184.00–528.10)	365.60 (227.40–583.60)	343.50 (198.60–567.00)	369 (221.00–502.30)
MMP-7 (ng/mL), median (IQR)	8.55 (5.60–11.55)	6.80 (4.74–10-78)	9.65 (5.53–12.90)	8.40 (5.73–12.05)	10.80 (5.05–13.85)
CA19-9 (U/mL), median (IQR)	18.70 (8.70–25.50)	16.15 (9.25–26.78)	16.00 (10.38–25.00)	16.45 (12.35–25.18)	19.40 (12.85–31.05)
CA-125 (U/mL), median (IQR)	6.20 (2.88–9.15)	4.90 (2.73–8.50)	5.45 (2.15–11.80)	5.35 (2.50–11.90)	6.25 (2.70–12.40)
CCL18 (ng/mL), mean (SD)	304.70 (87.15)	313.9 (73.12)	315.9 (88.44)	310.8 (87.41)	310.5 (85.00)
Periostin (ng/mL), median (IQR)	9.85 (6.10–20.00)	10.25 (6.10–15.23)	10.05 (6.80–13.13)	10.30 (7.18–16.78)	9 (6.30–14.80)

Notes: Baseline vs 12 months, *** $p < 0.001$, **** $p < 0.0001$; Baseline vs 18 months, ^ $p < 0.05$, ^^^^ $p < 0.0001$; Baseline vs 24 months, # $p < 0.05$, #### $p < 0.0001$; 6 months vs 12 months, §§ $p < 0.01$, §§§ $p < 0.001$; 6 months vs 18 months, •••• $p < 0.0001$; 6 months vs 24 months, ±± $p < 0.01$. **Abbreviations:** IPF – idiopathic pulmonary fibrosis, FEV_1 – expiratory volume in 1 second, FVC – forced vital capacity, $T_{L,CO}$ – transfer factor of the lung for carbon monoxide, 6MWT – six-minute walk test, KL-6 – Krebs von den Lungen-6, SP-D – surfactant protein D, MMP-7 – matrix metalloproteinase 7, CA19-9 – cancer antigen 19-9, CA-125 – cancer antigen 125, CCL18 – chemokine (C-C motif) ligand 18.

Table S7. Correlations of serum biomarkers levels with clinical measures in IPF patients at baseline.

	**FVC % pred.**	**$T_{L,CO}$ % pred.**	**Age (years)**	**Time since diagnosis (years)**	**Pack-years (years)**	**GAP index**	**CPI score**	**6MWT (meters)**
**KL-6 (U/mL)**	r = -0.67<br>*p* = 0.0001	r = -0.53<br>*p* = 0.004	r = -0.34<br>*p* = 0.07	r = 0.03<br>*p* = 0.89	r = 0.18<br>*p* = 0.36	r = 0.36<br>*p* = 0.06	r = 0.15<br>*p* = 0.44	r = -0.19<br>*p* = 0.34
**SP-D (ng/mL)**	r = -0.27<br>*p* = 0.16	r = -0.20<br>*p* = 0.30	r = -0.10<br>*p* = 0.60	r = -0.17<br>*p* = 0.38	r = 0.10<br>*p* = 0.62	r = 0.16<br>*p* = 0.41	r = -0.02<br>*p* = 0.91	r = 0.01<br>*p* = 0.97
**CA19-9 (U/mL)**	r = -0.09<br>*p* = 0.64	r = -0.34<br>*p* = 0.08	r = 0.06<br>*p* = 0.77	r = 0.45<br>*p* = 0.02	r = 0.15<br>*p* = 0.46	r = 0.33<br>*p* = 0.08	r = 0.31<br>*p* = 0.11	r = 0.23<br>*p* = 0.23
**CA-125 (U/mL)**	r = - 0.06<br>*p* = 0.75	r = -0.14<br>*p* = 0.46	r = 0.04<br>*p* = 0.84	r = 0.12<br>*p* = 0.56	r = 0.08<br>*p* = 0.68	r = 0.10<br>*p* = 0.60	r = 0.24<br>*p* = 0.23	r = 0.03<br>*p* = 0.87
**MMP-7 (ng/mL)**	r = -0.21<br>*p* = 0.27	r = -0.13<br>*p* = 0.51	r = 0.10<br>*p* = 0.63	r = -0.09<br>*p* = 0.63	r = -0.11<br>*p* = 0.57	r = -0.05<br>*p* = 0.78	r = -0.13<br>*p* = 0.53	r = -0.27<br>*p* = 0.17
**Periostin (ng/mL)**	r = -0.21<br>*p* = 0.27	r = -0.17<br>*p* = 0.39	r = -0.27<br>*p* = 0.17	r = -0.22<br>*p* = 0.26	r = 0.04<br>*p* = 0.83	r = 0.07<br>*p* = 0.74	r = -0.03<br>*p* = 0.87	r = -0.07<br>*p* = 0.73
**CCL18 (ng/mL)**	r = -0.17<br>*p* = 0.38	r = -0.38<br>*p* = 0.04	r = -0.12<br>*p* = 0.55	r = -0.18<br>*p* = 0.35	r = 0.20<br>*p* = 0.30	r = 0.31<br>*p* = 0.11	r = 0.44<br>*p* = 0.02	r = -0.08<br>*p* = 0.68

Abbreviations: IPF – idiopathic pulmonary fibrosis, FVC – forced vital capacity, $T_{L,CO}$ – transfer factor of the lung for carbon monoxide, 6MWT – six-minute walk test, KL-6 – Krebs von den Lungen-6, SP-D – surfactant protein D, CA19-9 – cancer

antigen 19-9, CA-125 – cancer antigen 125, MMP-7 – matrix metalloproteinase 7, CCL18 – chemokine (C-C motif) ligand 18.

Table S8. Correlations of serum biomarkers levels with clinical measures in IPF patients after 6 months of antifibrotic therapy.

	**FVC % pred.**	**$T_{L,CO}$ % pred.**	**Age (years)**	**Time since diagnosis (years)**	**Pack-years (years)**	**GAP index**	**CPI score**	**6MWT (meters)**
**KL-6 (U/mL)**	r = -0.57<br>*p* = 0.002	r = -0.46<br>*p* = 0.02	r = -0.40<br>*p* = 0.03	r = -0.08<br>*p* = 0.67	r = 0.11<br>*p* = 0.59	r = 0.16<br>*p* = 0.43	r = 0.23<br>*p* = 0.23	r = -0.21<br>*p* = 0.34
**SP-D (ng/mL)**	r = -0.25<br>*p* = 0.19	r = -0.15<br>*p* = 0.45	r = -0.12<br>*p* = 0.53	r = -0.16<br>*p* = 0.42	r = 0.08<br>*p* = 0.69	r = 0.12<br>*p* = 0.53	r = -0.19<br>*p* = 0.33	r = 0.04<br>*p* = 0.85
**CA19-9 (U/mL)**	r = 0.02<br>*p* = 0.94	r = -0.39<br>*p* = 0.04	r = 0.05<br>*p* = 0.80	r = 0.39<br>*p* = 0.04	r = 0.14<br>*p* = 0.49	r = 0.23<br>*p* = 0.24	r = 0.41<br>*p* = 0.03	r = 0.32<br>*p* = 0.14
**CA-125 (U/mL)**	r = -0.07<br>*p* = 0.74	r = -0.34<br>*p* = 0.09	r = 0.14<br>*p* = 0.48	r = 0.23<br>*p* = 0.23	r = 0.06<br>*p* = 0.78	r = 0.26<br>*p* = 0.18	r = 0.50<br>*p* = 0.007	r = -0.29<br>*p* = 0.18
**MMP-7 (ng/mL)**	r = -0.08<br>*p* = 0.70	r = -0.06<br>*p* = 0.76	r = 0.05<br>*p* = 0.80	r = 0.14<br>*p* = 0.47	r = -0.15<br>*p* = 0.45	r = 0.05<br>*p* = 0.81	r = -0.02<br>*p* = 0.92	r = 0.01<br>*p* = 0.98
**Periostin (ng/mL)**	r = -0.15<br>*p* = 0.44	r = -0.31<br>*p* = 0.12	r = -0.33<br>*p* = 0.09	r = 0.09<br>*p* = 0.65	r = 0.002<br>*p* = 0.99	r = 0.29<br>*p* = 0.14	r = 0.18<br>*p* = 0.35	r = 0.14<br>*p* = 0.51
**CCL18 (ng/mL)**	r = -0.16<br>*p* = 0.40	r = -0.30<br>*p* = 0.13	r = -0.22<br>*p* = 0.27	r = 0.01<br>*p* = 0.97	r = 0.33<br>*p* = 0.09	r = 0.20<br>*p* = 0.31	r = 0.45<br>*p* = 0.02	r = -0.20<br>*p* = 0.36

Abbreviations: IPF – idiopathic pulmonary fibrosis, FVC – forced vital capacity, $T_{L,CO}$ – transfer factor of the lung for carbon monoxide, 6MWT – six-minute walk test, KL-6 – Krebs von den Lungen-6, SP-D – surfactant protein D, CA19-9 – cancer antigen 19-9, CA-125 – cancer antigen 125, MMP-7 – matrix metalloproteinase 7, CCL18 – chemokine (C-C motif) ligand 18.

Table S9. Correlations of serum biomarkers levels with clinical measures in IPF patients after 12 months of antifibrotic therapy.

	**FVC % pred.**	**$T_{L,CO}$ % pred.**	**Age (years)**	**Time since diagnosis (years)**	**Pack-years (years)**	**GAP index**	**CPI score**	**6MWT (meters)**
**KL-6 (U/mL)**	r = -0.60<br>*p* = 0.0008	r = -0.48<br>*p* = 0.0098	r = -0.25<br>*p* = 0.19	r = -0.01<br>*p* = 0.96	r = 0.10<br>*p* = 0.61	r = 0.33<br>*p* = 0.09	r = 0.27<br>*p* = 0.17	r = -0.36<br>*p* = 0.06
**SP-D (ng/mL)**	r = -0.33<br>*p* = 0.09	r = -0.29<br>*p* = 0.14	r = -0.10<br>*p* = 0.60	r = -0.09<br>*p* = 0.66	r = 0.13<br>*p* = 0.49	r = 0.27<br>*p* = 0.17	r = 0.11<br>*p* = 0.58	r = -0.23<br>*p* = 0.24
**CA19-9 (U/mL)**	r = 0.01<br>*p* = 0.95	r = -0.37<br>*p* = 0.05	r = 0.09<br>*p* = 0.65	r = 0.36<br>*p* = 0.06	r = 0.05<br>*p* = 0.81	r = 0.29<br>*p* = 0.13	r = 0.39<br>*p* = 0.04	r = -0.05<br>*p* = 0.78
**CA-125 (U/mL)**	r = -0.04<br>*p* = 0.82	r = -0.11<br>*p* = 0.59	r = 0.06<br>*p* = 0.76	r = 0.22<br>*p* = 0.27	r = 0.02<br>*p* = 0.90	r = 0.19<br>*p* = 0.34	r = 0.24<br>*p* = 0.22	r = -0.11<br>*p* = 0.58
**MMP-7 (ng/mL)**	r = 0.004<br>*p* = 0.98	r = -0.22<br>*p* = 0.26	r = 0.03<br>*p* = 0.89	r = -0.06<br>*p* = 0.78	r = -0.17<br>*p* = 0.38	r = -0.03<br>*p* = 0.89	r = 0.13<br>*p* = 0.52	r = -0.14<br>*p* = 0.47
**Periostin (ng/mL)**	r = -0.17<br>*p* = 0.39	r = -0.22<br>*p* = 0.27	r = -0.37<br>*p* = 0.05	r = -0.19<br>*p* = 0.32	r = -0.02<br>*p* = 0.92	r = 0.10<br>*p* = 0.63	r = 0.13<br>*p* = 0.52	r = 0,02<br>*p* = 0.93
**CCL18 (ng/mL)**	r = -0.06<br>*p* = 0.76	r = -0.21<br>*p* = 0.28	r = -0.07<br>*p* = 0.71	r = 0.12<br>*p* = 0.54	r = 0.07<br>*p* = 0.74	r = 0.29<br>*p* = 0.13	r = 0.42<br>*p* = 0.03	r = -0.12<br>*p* = 0.53

Abbreviations: IPF – idiopathic pulmonary fibrosis, FVC – forced vital capacity, $T_{L,CO}$ – transfer factor of the lung for carbon monoxide, 6MWT – six-minute walk test, KL-6 – Krebs von den Lungen-6, SP-D – surfactant protein D, CA19-9 – cancer antigen 19-9, CA-125 – cancer antigen 125, MMP-7 – matrix metalloproteinase 7, CCL18 – chemokine (C-C motif) ligand 18.

Table S10. Correlations of serum biomarkers levels with clinical measures in IPF patients after 18 months of antifibrotic therapy.

	FVC % pred.	$T_{L,CO}$ % pred.	Age (years)	Time since diagnosis (years)	Pack-years (years)	GAP index	CPI score	6MWT (meters)
**KL-6 (U/mL)**	r = -0.41<br>*p* = 0.03	r = -0.53<br>*p* = 0.004	r = -0.20<br>*p* = 0.31	r = -0.02<br>*p* = 0.93	r = 0.18<br>*p* = 0.35	r = 0.26<br>*p* = 0.18	r = 0.46<br>*p* = 0.01	r = -0.28<br>*p* = 0.32
**SP-D (ng/mL)**	r = -0.34<br>*p* = 0.08	r = -0.29<br>*p* = 0.14	r = -0.03<br>*p* = 0.88	r = -0.15<br>*p* = 0.46	r = 0.10<br>*p* = 0.60	r = 0.25<br>*p* = 0.20	r = 0.24<br>*p* = 0.22	r = -0.01<br>*p* = 0.95
**CA19-9 (U/ml)**	r = -0.11<br>*p* = 0.59	r = -0.33<br>*p* = 0.09	r = 0.03<br>*p* = 0.90	r = 0.42<br>*p* = 0.03	r = 0.09<br>*p* = 0.63	r = 0.24<br>*p* = 0.23	r = 0.31<br>*p* = 0.10	r = 0.19<br>*p* = 0.35
**CA-125 (U/mL)**	r = 0.13<br>*p* = 0.51	r = -0.14<br>*p* = 0.48	r = 0.10<br>*p* = 0.60	r = 0.22<br>*p* = 0.26	r = 0.08<br>*p* = 0.70	r = 0.16<br>*p* = 0.42	r = 0.22<br>*p* = 0.26	r = -0.17<br>*p* = 0.41
**MMP-7 (ng/mL)**	r = -0.09<br>*p* = 0.64	r = -0.25<br>*p* = 0.21	r = 0.09<br>*p* = 0.66	r = -0.02<br>*p* = 0.91	r = -0.24<br>*p* = 0.23	r = 0.22<br>*p* = 0.25	r = 0.12<br>*p* = 0.55	r = 0.01<br>*p* = 0.95
**Periostin (ng/mL)**	r = -0.20<br>*p* = 0.31	r = -0.19<br>*p* = 0.34	r = -0.24<br>*p* = 0.22	r = -0.09<br>*p* = 0.64	r = -0.15<br>*p* = 0.45	r = 0.02<br>*p* = 0.92	r = -0.05<br>*p* = 0.81	r = -0.18<br>*p* = 0.39
**CCL18 (ng/mL)**	r = 0.04<br>*p* = 0.86	r = -0.10<br>*p* = 0.62	r = -0.23<br>*p* = 0.24	r = -0.03<br>*p* = 0.90	r = 0.27<br>*p* = 0.17	r = 0.03<br>*p* = 0.89	r = 0.12<br>*p* = 0.53	r = -0.19<br>*p* = 0.35

Abbreviations: IPF – idiopathic pulmonary fibrosis, FVC – forced vital capacity, $T_{L,CO}$ – transfer factor of the lung for carbon monoxide, 6MWT – six-minute walk test, KL-6 – Krebs von den Lungen-6, SP-D – surfactant protein D, CA19-9 – cancer antigen 19-9, CA-125 – cancer antigen 125, MMP-7 – matrix metalloproteinase 7, CCL18 – chemokine (C-C motif) ligand 18.

Table S11. Correlations of serum biomarkers levels with clinical measures in IPF patients after 24 months of antifibrotic therapy.

	FVC % pred.	$T_{L,CO}$ % pred.	Age (years)	Time since diagnosis (years)	Pack-years (years)	GAP index	CPI score	6MWT (meters)
**KL-6 (U/mL)**	r = -0.50<br>*p* = 0.006	r = -0.63<br>*p* = 0.0006	r = -0.42<br>*p* = 0.03	r = 0.21<br>*p* = 0.28	r = 0.14<br>*p* = 0.48	r = 0.27<br>*p* = 0.16	r = 0.33<br>*p* = 0.08	r = -0.27<br>*p* = 0.21
**SP-D (ng/mL)**	r = -0.41<br>*p* = 0.03	r = -0.47<br>*p* = 0.02	r = -0.22<br>*p* = 0.27	r = -0.04<br>*p* = 0.84	r = 0.14<br>*p* = 0.48	r = 0.43<br>*p* = 0.02	r = 0.11<br>*p* = 0.57	r = -0.32<br>*p* = 0.12
**CA19-9 (U/mL)**	r = 0.001<br>*p* = 0.99	r = -0.48<br>*p* = 0.01	r = -0.0003<br>*p* = 0.99	r = 0.45<br>*p* = 0.02	r = 0.11<br>*p* = 0.57	r = 0.26<br>*p* = 0.19	r = 0.56<br>*p* = 0.002	r = -0.14<br>*p* = 0.51
**CA-125 (U/mL)**	r = -0.04<br>*p* = 0.86	r = -0.10<br>*p* = 0.63	r = 0.16<br>*p* = 0.43	r = 0.24<br>*p* = 0.21	r = -0.001<br>*p* = 0.99	r = 0.17<br>*p* = 0.38	r = 0.28<br>*p* = 0.16	r = -0.009<br>*p* = 0.97
**MMP-7 (ng**	r = 0.04<br>*p* = 0.82	r = -0.07<br>*p* = 0.72	r = 0.10<br>*p* = 0.62	r = 0.04<br>*p* = 0.82	r = -0.14<br>*p* = 0.48	r = -0.12<br>*p* = 0.56	r = 0.09<br>*p* = 0.63	r = -0.02<br>*p* = 0.92
**Periostin (ng/mL)**	r = -0.25<br>*p* = 0.22	r = -0.16<br>*p* = 0.45	r = -0.29<br>*p* = 0.15	r = -0.12<br>*p* = 0.57	r = -0.12<br>*p* = 0.54	r = -0.06<br>*p* = 0.78	r = 0.0002<br>*p* = 0.99	r = 0.22<br>*p* = 0.31
**CCL18 (ng/mL)**	r = -0.19<br>*p* = 0.34	r = -0.48<br>*p* = 0.01	r = -0.12<br>*p* = 0.54	r = -0.16<br>*p* = 0.40	r = 0.20<br>*p* = 0.32	r = 0.42<br>*p* = 0.03	r = 0.40<br>*p* = 0.03	r = -0.23<br>*p* = 0.27

Abbreviations: IPF – idiopathic pulmonary fibrosis, FVC – forced vital capacity, $T_{L,CO}$ – transfer factor of the lung for carbon monoxide, 6MWT – six-minute walk test, KL-6 – Krebs von den Lungen-6, SP-D – surfactant protein D, CA19-9 – cancer antigen 19-9, CA-125 – cancer antigen 125, MMP-7 – matrix metalloproteinase 7, CCL18 – chemokine (C-C motif) ligand 18.

Table S12. Associations of changes in serum biomarkers levels and changes in physiologic and functional measures in the stables subgroup of patients with IPF (n = 18) during the first year of antifibrotic therapy.

	Change in FVC % pred. (absolute change)	Change in $T_{L,CO}$ % pred. (absolute change)	Change in 6MWT (%)
**Change in KL-6 serum levels (%)**	r = 0.40<br>*p* = 0.10	r = 0.21<br>*p* = 0.41	r = -0.20<br>*p* = 0.44
**Change in SP-D serum levels (%)**	r = 0.34<br>*p* = 0.17	r = -0.29<br>*p* = 0.24	r = -0.38<br>*p* = 0.12
**Change in CA19-9 serum levels (%)**	r = -0.09<br>*p* = 0.74	r = -0.34<br>*p* = 0.20	r = -0.002<br>*p* = 0.99
**Change in CA-125 serum levels (%)**	r = -0.11<br>*p* = 0.65	r = 0.01<br>*p* = 0.96	r = -0.37<br>*p* = 0.13
**Change in MMP-7 serum levels (%)**	r = 0.09<br>*p* = 0.71	r = -0.06<br>*p* = 0.82	r = -0.19<br>*p* = 0.45
**Change in periostin serum levels (%)**	r = -0.01<br>*p* = 0.96	r = 0.47<br>*p* = 0.05	r = -0.01<br>*p* = 0.96
**Change in CCL18 serum levels (%)**	r = -0.03<br>*p* = 0.92	r = 0.09<br>*p* = 0.73	r = -0.04<br>*p* = 0.86

Table S13. Associations of changes in serum biomarkers levels and changes in physiologic and functional measures in the progressors subgroup of patients with IPF (n = 10) during the first year of antifibrotic therapy.

	Change in FVC % pred. (absolute change)	Change in $T_{L,CO}$ % pred. (absolute change)	Change in 6MWT (%)
**Change in KL-6 serum levels (%)**	r = -0.62<br>*p* = 0.06	r = -0.16<br>*p* = 0.66	r = 0.28<br>*p* = 0.43
**Change in SP-D serum levels (%)**	r = -0.72<br>*p* = 0.02	r = -0.19<br>*p* = 0.61	r = 0.12<br>*p* = 0.76
**Change in CA19-9 serum levels (%)**	r = 0.49<br>*p* = 0.15	r = -0.16<br>*p* = 0.66	r = 0.01<br>*p* = 0.99
**Change in CA-125 serum levels (%)**	r = 0.21<br>*p* = 0.56	r = 0.05<br>*p* = 0.89	r = 0.02<br>*p* = 0.97
**Change in MMP-7 serum levels (%)**	r = 0.20<br>*p* = 0.58	r = -0.21<br>*p* = 0.56	r = -0.53<br>*p* = 0.12
**Change in periostin serum levels (%)**	r = 0.09<br>*p* = 0.81	r = -0.28<br>*p* = 0.43	r = 0.01<br>*p* = 0.99
**Change in CCL18 serum levels (%)**	r = -0.27<br>*p* = 0.45	r = -0.36<br>*p* = 0.31	r = 0.83<br>*p* = 0.005

Table S14. Associations of changes in serum biomarkers levels and changes in physiologic and functional measures in the stables subgroup of patients with IPF (n = 17) during the second year of antifibrotic therapy.

	Change in FVC % pred. (absolute change)	Change in $T_{L,CO}$ % pred. (absolute change)	Change in 6MWT (%)
**Change in KL-6 serum levels (%)**	r = 0.49<br>*p* = 0.05	r = 0.10<br>*p* = 0.72	r = -0.26<br>*p* = 0.39
**Change in SP-D serum levels (%)**	r = 0.16<br>*p* = 0.54	r = -0.09<br>*p* = 0.75	r = 0.06<br>*p* = 0.85
**Change in CA19-9 serum levels (%)**	r = 0.09<br>*p* = 0.75	r = 0.04<br>*p* = 0.88	r = 0.08<br>*p* = 0.80

**Change in CA-125 serum levels (%)**	r = -0.01<br>*p* = 0.96	r = 0.00<br>*p* = 0.99	r = 0.19<br>*p* = 0.53
**Change in MMP-7 serum levels (%)**	r = -0.06<br>*p* = 0.82	r = -0.29<br>*p* = 0.27	r = -0.16<br>*p* = 0.59
**Change in periostin serum levels (%)**	r = -0.17<br>*p* = 0.51	r = -0.21<br>*p* = 0.44	r = 0.08<br>*p* = 0.81
**Change in CCL18 serum levels (%)**	r = 0.35<br>*p* = 0.17	r = -0.44<br>*p* = 0.09	r = 0.33<br>*p* = 0.27

Table S15. Associations of changes in serum biomarkers levels and changes in physiologic and functional measures in the progressors subgroup of patients with IPF (n = 11) during the second year of antifibrotic therapy.

	**Change in FVC % pred. (absolute change)**	**Change in $T_{L,CO}$ % pred. (absolute change)**	**Change in 6MWT (%)**
**Change in KL-6 serum levels (%)**	r = -0.71<br>*p* = 0.02	r = -0.12<br>*p* = 0.76	r = 0.20<br>*p* = 0.56
**Change in SP-D serum levels (%)**	r = -0.06<br>*p* = 0.86	r = -0.22<br>*p* = 0.54	r = -0.16<br>*p* = 0.63
**Change in CA19-9 serum levels (%)**	r = -0.23<br>*p* = 0.55	r = 0.17<br>*p* = 0.70	r = 0.13<br>*p* = 0.74
**Change in CA-125 serum levels (%)**	r = -0.25<br>*p* = 0.47	r = -0.49<br>*p* = 0.15	r = 0.41<br>*p* = 0.21
**Change in MMP-7 serum levels (%)**	r = -0.14<br>*p* = 0.69	r = 0.08<br>*p* = 0.84	r = -0.13<br>*p* = 0.71
**Change in periostin serum levels (%)**	r = -0.45<br>*p* = 0.17	r = -0.59<br>*p* = 0.08	r = 0.28<br>*p* = 0.40
**Change in CCL18 serum levels (%)**	r = -0.12<br>*p* = 0.73	r = 0.05<br>*p* = 0.89	r = 0.21<br>*p* = 0.54
